# Modulation of the miR-485-3p/PGC-1α Pathway by ASO-Loaded Nanoparticles Attenuates ALS Pathogenesis

**DOI:** 10.3390/ijms27020615

**Published:** 2026-01-07

**Authors:** In Soo Ryu, Dae-In Ha, Yeon-Joo Jung, Hyo Jin Lee, Insun Kim, Yu Na Lim, Hyun Su Min, Seung Hyun Kim, Ilsang Yoon, Hyun-Jeong Cho, Jin-Hyeob Ryu

**Affiliations:** 1 BIORCHESTRA Co., Ltd., 1, Gukjegwahak 2-ro, Yuseong-gu, Daejeon 34000, Republic of Korea; insooryu@biorchestra.com (I.S.R.); yjjung@biorchestra.com (Y.-J.J.); hohosaraly@biorchestra.com (H.J.L.); inssony@biorchestra.com (I.K.); isyoon@biorchestra.com (I.Y.); 2 Department of Neurology, College of Medicine, Hanyang University, 222, Wangsimni-ro, Seongdong-gu, Seoul 04763, Republic of Korea; kimsh1@hanyang.ac.kr; 3 Department of Biomedical Laboratory Science, College of Medical Science, Konyang University, 158 Gwanjeodong-ro, Seo-gu, Daejeon 35365, Republic of Korea

**Keywords:** nanoparticle, antisense nucleotide (ASO), neurodegenerative disease, microRNA, neuroinflammation

## Abstract

Amyotrophic lateral sclerosis (ALS) is a fatal neurodegenerative disorder characterized by progressive motor neuron degeneration with limited treatment options. In this study, we investigated the pathological role of microRNA-485-3p (miR-485-3p) in ALS, particularly its regulation of PGC-1α, a transcriptional coactivator essential for mitochondrial function and neuroprotection. We also evaluated the therapeutic potential of BMD-001S, a nanoparticle-based formulation encapsulating an antisense oligonucleotide targeting miR-485-3p. Our results demonstrated that miR-485-3p expression was significantly elevated in both SOD1^G93A^-expressing HMC3 microglial cells and in the spinal cords of SOD1^G93A^ transgenic mice at late disease stages, implicating its contribution to ALS pathogenesis. Intravenous administration of BMD-001S effectively reduced miR-485-3p levels and restored *PGC-1α* mRNA and PGC-1α protein expression in the spinal cord. These molecular changes were associated with notable therapeutic outcomes, including reduced SOD1 protein aggregation, decreased neuroinflammation, and lower neurofilament light chain concentrations in cerebrospinal fluid. Moreover, BMD-001S treatment was associated with improvements in electrophysiological parameters and preservation of neuromuscular junction integrity during the observation period in SOD1^G93A^ transgenic mice. Taken together, these findings suggest that miR-485-3p/PGC-1α pathway is a promising therapeutic target in ALS and support the potential of BMD-001S as a novel treatment strategy for the disease.

## 1. Introduction

Amyotrophic lateral sclerosis (ALS) is a fatal neurodegenerative disorder characterized by the progressive degeneration of motor neurons in the brain and spinal cord, ultimately resulting in muscle weakness, paralysis, and respiratory failure [1,2]. ALS is considered part of a broader spectrum of motor neuron diseases, distinguished by the selective vulnerability of both upper and lower motor neurons [3,4]. Pathologically, ALS is marked by the accumulation of misfolded proteins, mitochondrial dysfunction, oxidative stress, and inflammation, all of which contribute to neuronal death. While the majority of ALS cases are sporadic, approximately 10% are familial, most frequently associated with mutations in genes such as superoxide dismutase 1 (SOD1), C9orf72, and TAR DNA-binding protein 43 [5,6,7]. Despite significant advances in elucidating the genetic and molecular mechanisms underlying ALS, current therapeutic options remain limited Existing treatments primarily focus on symptom management and modestly improving quality of life, with survival benefits typically extending only a few months [8,9]. Genetic therapies, such as antisense oligonucleotides (ASOs), offer promise by targeting specific disease-associated genes; for example, the ASO tofersen, which targets SOD1 mutations, has demonstrated target engagement, reduction in SOD1 protein levels, and modulation of neurofilament light chain (NfL) biomarkers in phase 1–2 and phase 3 clinical trials in patients with SOD1-associated ALS [10,11]. However, the broader clinical application of ASO-based therapies remains challenged by delivery barriers, potential off-target effects, and limited efficacy across the heterogeneous genotypes of ALS [10,11,12,13].

Emerging studies demonstrate that microRNAs (miRNAs) are intimately involved in brain development and the maintenance of biological functions within the central nervous system (CNS) [14,15,16]. Various miRNAs have been investigated as therapeutics and biomarkers for disease progression and diagnosis in CNS disorders, including ALS [17,18,19]. Several studies have identified differentially expressed miRNAs in ALS patients compared to healthy controls and individuals with other neurological disorders. For example, Raheja and colleagues reported 7 upregulated miRNAs (including miR-192-5p, miR-1, and miR-133a-3p) and 6 downregulated miRNAs (including miR-320c and miR-320a) in the serum of ALS patients [20]. Furthermore, Emde and colleagues suggested that global dysregulation of miRNAs may be a common feature across various ALS subtypes [21]. Collectively, these findings underscore the potential of miRNAs as diagnostic and prognostic biomarkers for ALS and highlight their possible involvement in disease progression, thereby opening new avenues for therapeutic interventions.

Among these, miR-485-3p has garnered considerable attention for its involvement in various neurodegenerative conditions, including ALS. MiR-485-3p has been implicated in the regulation of neuroinflammation, synaptic plasticity, and mitochondrial function, all of which are disrupted in ALS pathology [22,23]. A critical target of miR-485-3p is peroxisome proliferator-activated receptor gamma coactivator 1-alpha (PGC-1α), a master regulator of mitochondrial biogenesis and oxidative stress responses, whose 3′-UTR has been experimentally validated as a direct miR-485-3p binding site by Lou et al. using luciferase reporter assays, and is also consistently predicted as a conserved target by in silico analyses such as TargetScan [24]. PGC-1α regulates mitochondrial biogenesis, cellular respiration, and energy metabolism, and demonstrated therapeutic potential in ALS. Studies have shown that transgenic overexpression of PGC-1α improves motor neuron function, extends lifespan, and reduces cellular stress by enhancing mitochondrial biogenesis and function in ALS mouse models [25]. Additionally, decreased mRNA expression of PGC-1α and its associated factors have been observed in both the SOD1^G93A^ transgenic mouse model and human sporadic ALS cases [26]. These findings suggest that reduced PGC-1α activity may contribute to ALS pathology, and that enhancing its expression could mitigate disease mechanisms. Despite the potential association between dysregulated miR-485-3p expression and abnormal PGC-1α expression patterns in ALS, no studies to date have directly investigated this relationship.

In this study, we aimed to investigate the role of miR-485-3p in the pathology of ALS using both in vitro and in vivo models, and to evaluate its potential as a therapeutic target. We hypothesized that modulating miR-485-3p could influence ALS progression by regulating PGC-1α activity. To explore this hypothesis, we developed BMD-001S, a nanoparticle-based platform (BDDS™) that targets the L-type amino acid transporter 1 (LAT1) and incorporates a modified antisense oligonucleotide (ASO) against miR-485-3p. Our research focused on determining whether BMD-001S could effectively modulate miR-485-3p-mediated regulation of PGC-1α activity during ALS progression and improve motor neuron function in an ALS mouse model. These findings suggest that BMD-001S may represent a promising therapeutic option for ALS, providing a novel strategy to improve treatment outcomes for patients with this disease.

## 2. Results

### 2.1. Dysregulation of miR-485-3p in SOD1^G93A^-Expressing HMC3 Cells

**Figure 1:**
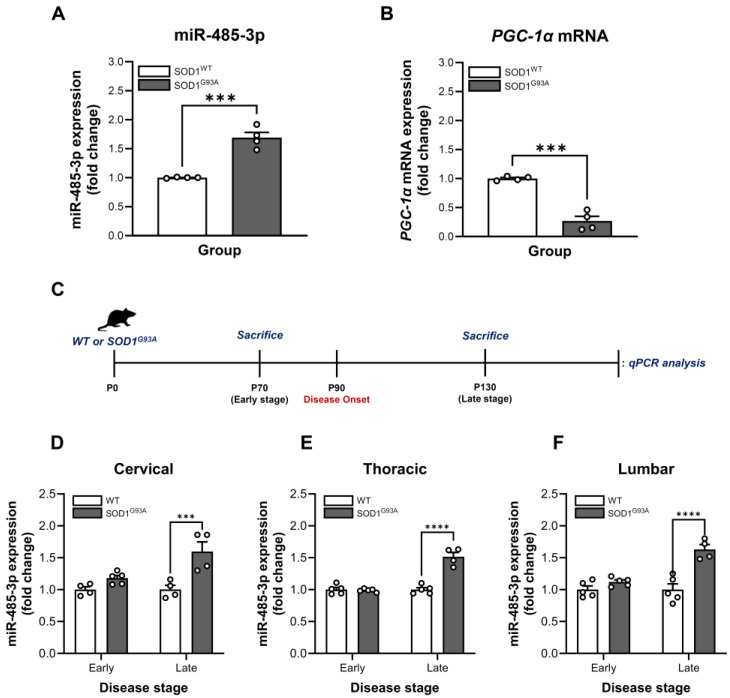
Dysregulated expressions of miR-485-3p and PGC-1α by SOD1^G93A^ expression in in vitro and in vivo models. (**A**) Expression level changes in miR-485-3p by SOD1^G93A^ expression in HMC3 cell line. (**B**) Abnormal expression of *PGC-1α* mRNA in SOD1^G93A^-expressing cells. (**C**) Experimental timeline for disease progression and analysis in SOD1^G93A^ mouse model. The level of miR-485-3p was compared at early stage (P70) and late stage (P130). Comparative miR-485-3p expression changes in the (**D**) Cervical, (**E**) Thoracic, and (**F**) Lumbar spinal cord regions. Data are presented as mean ± SEM. *** *p* < 0.001, **** *p* < 0.0001 vs. SOD1^WT^-expressing cells in vitro and the WT group in vivo, respectively; n = 4–5 per group.

To investigate the role of miR-485-3p in ALS, we first established stable human microglial clone 3 (HMC3) cell lines expressing either wild-type *SOD1* (*SOD1*^WT^) or mutant *SOD1*^G93A^ (Supplementary Figure S1). Expression analysis revealed that miR-485-3p levels were significantly increased in SOD1^G93A^-expressing HMC3 cells compared to SOD1^WT^-expressing cells, with a 1.7-fold upregulation (unpaired *t*-test, *t*_(6)_ = 7.482, *p* = 0.0003) (Figure 1A). Furthermore, mRNA expression of PGC-1α, a putative target of miR-485-3p [24], was markedly decreased in SOD1^G93A^-expressing HMC3 cells, showing an approximately 75% reduction relative to SOD1^WT^-expressing cells (unpaired *t*-test, *t*_(6)_ = 8.868, *p* = 0.001) (Figure 1B).

### 2.2. Stage- and Region-Specific Alterations of miR-485-3p Expression in SOD1^G93A^ Transgenic Mice

Based on these in vitro findings, we further examined the relationship between miR-485-3p and ALS disease progression in vivo. Specifically, we analyzed miR-485-3p expression in spinal cord tissues at early and late stages of disease in both WT and SOD1^G93A^ transgenic mice, as these regions are critically implicated in the pathophysiology of ALS [27,28,29]. Given that SOD1^G93A^ transgenic mice typically exhibit disease onset—characterized by hindlimb tremors—around 90 days of age [30,31,32,33,34,35], we assessed miR-485-3p expression in the spinal cord at 70 days (early stage) and 130 days (late stage) using quantitative PCR (qPCR) (Figure 1C). At the late stage, SOD1^G93A^ mice demonstrated a 1.6-fold increase in miR-485-3p expression in the cervical spinal cord relative to WT controls (Stage: *F*_(1,13)_ = 6.173, *p* = 0.0272; Genotype: *F*_(1,13)_ = 21.09, *p* = 0.0005; Interaction: *F*_(1,13)_ = 6.047, *p* = 0.0287) (Figure 1D). Consistently, the thoracic and lumbar spinal cord regions of SOD1^G93A^ mice also exhibited elevated miR-485-3p levels—1.5-fold and 1.6-fold increases, respectively—compared to WT mice at the same stage (thoracic spinal cord, Stage: *F*_(1,15)_ = 50.74, *p* < 0.0001; Genotype: *F*_(1,15)_ = 46.21, *p* < 0.0001; Interaction: *F*_(1,15)_ = 51.52, *p* < 0.0001; lumbar spinal cord, Stage: *F*_(1,15)_ = 14.98, *p* = 0.0015; Genotype: *F*_(1,15)_ = 32.47, *p* < 0.0001; Interaction: *F*_(1,15)_ = 14.98, *p* = 0015) (Figure 1E,F). However, no statistically significant differences in miR-485-3p expression were observed between genotypes at the early stage in the cervical, thoracic, and lumbar spinal cord regions (Figure 1D–F).

### 2.3. Characterization of BMD-001S

BMD-001S exhibited a size approximately 44.6 ± 5.5 nm and a polydispersity index (PDI) of 0.14 ± 0.04, indicating a uniform nanoparticle size distribution. The zeta potential was measured as 8.3 ± 1.4 mV, and the pH of the formulation was 7.58 ± 0.13. The encapsulation efficiency (EE%) of the ASO was 98.4 ± 3.5%, demonstrating efficient ASO loading. These characterization data for BMD-001S are provided in Supplementary Table S1.

### 2.4. Cytotoxicity and Pharmacological Effect of BMD-001S in SOD1^G93A^-Expressing HMC3 Cells

**Figure 2:**
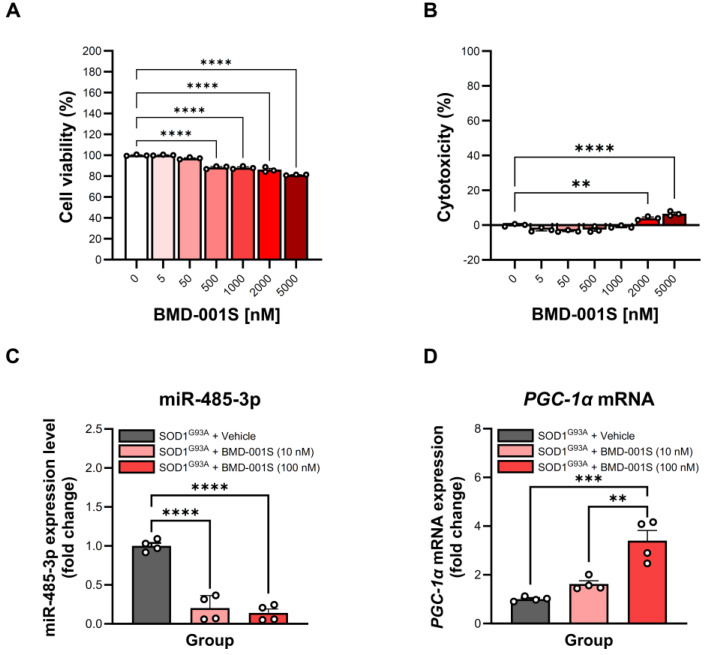
Toxicity and efficacy assessment of BMD-001S in HMC3 cells stably expressing SOD1^G93A^. (**A**,**B**) Determination of cell viability and cytotoxicity in SOD1^G93A^-expressing cells after treatment of BMD-001S at concentrations ranging from 0 to 5000 nM. Each value was calculated based on vehicle control (white bar). (**C**) Regulation of miR-485-3p with a treatment of 10 and 100 nM of BMD-001S. (**D**) Recovery of PGC-1α originated from the knockdown of miR-485-3p with a treatment of 10 and 100 nM of BMD-001S. Each level was calculated by comparing vehicle condition as a control. Data are presented as mean ± SEM. ** *p* < 0.01, *** *p* < 0.001, **** *p* < 0.0001 vs. the vehicle control; n = 3–4 per group.

For nanoparticles encapsulating ASO to be viable therapeutic agents, they must exhibit minimal cytotoxicity alongside demonstrable pharmacological efficacy, such as target gene knockdown [36,37,38]. To evaluate the cytotoxicity BMD-001S, we assessed cell viability and cytotoxicity in SOD1^G93A^-expressing HMC3 cells. The results showed that treatment with BMD-001S at concentrations of 500, 1000, 2000, and 5000 nM resulted in a modest but statistically significant decrease in cell viability compared to vehicle-treated controls (*F*_(6,14)_ = 108.9, *p* < 0.0001) (Figure 2A). However, no significant changes were observed at lower concentration of 5 and 50 nM (Figure 2A). Similarly, cytotoxicity was significantly elevated at 2000 and 5000 nM, relative to controls (*F*_(6,14)_ = 35.77, *p* < 0.0001) (Figure 2B). Based on these results, BMD-001S concentration exceeding 500 nM were considered cytotoxic in the SOD1^G93A^-expressing HMC3 cells; therefore, subsequent pharmacological evaluations were conducted using BMD-001S at concentrations below 500 nM.

To investigate the pharmacological effects of BMD-001S, we examined the modulation of the dysregulated miR-485-3p and its downstream target, *PGC-1α* mRNA, in SOD1^G93A^-expressing HMC3 cells 24 h following treatment with 10 or 100 nM of BMD-001S. Treatment with both concentrations (10 and 100 nM) resulted in a significant reduction in miR-485-3p expression compared to vehicle-treated controls (*F*_(2,9)_ = 67.13, *p* < 0.0001) (Figure 2C). Furthermore, *PGC-1α* mRNA levels were significantly upregulated in a dose-dependent manner (*F*_(2,9)_ = 23.33, *p* = 0.0003) (Figure 2D). These results indicate that BMD-001S effectively counteracts miR-485-3p-mediated suppression of *PGC-1α* mRNA.

### 2.5. BMD-001S Enhanced PGC-1α Expression in the Spinal Cord Regions of SOD1^G93A^ Transgenic Mice

**Figure 3:**
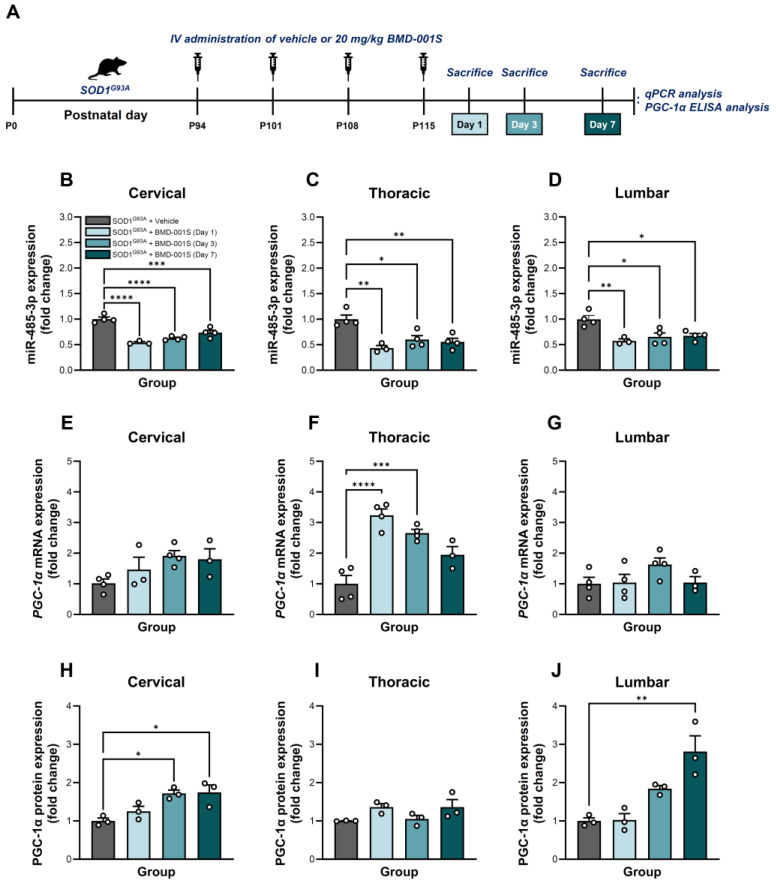
Biomolecular therapeutic effect of BMD-001S in SOD1^G93A^ transgenic mouse model. (**A**) Schematic procedure to evaluate the effect of BMD-001S in SOD1^G93A^ mouse model. The molecular biological changes in spinal cord tissues and CSF were analyzed at 1 day (light green), 3 days (intermediate green), and 7 days (green) following four IV injections of 20 mg/kg each, administered at one-week intervals. (**B**–**D**) Changes in expression level of miR-485-3p evaluated by qPCR compared to the vehicle-treated control. (**E**–**J**) Changes in *PGC-1α* mRNA and protein expression levels in the spinal cord. Data are presented as mean ± SEM. * *p* < 0.05, ** *p* < 0.01, *** *p* < 0.001, **** *p* < 0.0001 vs. the vehicle-treated control; n = 3–4 per group.

To investigate biomolecular restoration effect of BMD-001S in vivo, we administered intravenous (IV) injections of BMD-001S at a dose of 20 mg/kg to SOD1^G93A^ mice following established ALS symptom onset, with four weekly doses (Figure 3A). At one day after the final injection of BMD-001S, miR-485-3p levels were significantly reduced in all regions of the spinal cord compared to vehicle-treated controls. Specifically, miR-485-3p expression was significantly decreased by 50% in the cervical spinal cord (*F*_(3,11)_ = 32.18, *p* < 0.0001; Figure 3B), by 60% in the thoracic spinal cord (*F*_(3,11)_ = 10.66, *p* = 0.0014; Figure 3C), and by 50% in lumbar spinal cord (*F*_(3,11)_ = 8.545, *p* = 0.0033; Figure 3D). Over the subsequent days, miR-485-3p levels in all regions of spinal cord gradually increased but remained below those observed in the vehicle control group by day 7 post-final injection (Figure 3B–D).

In parallel, analysis of *PGC-1α* mRNA expression revealed a non-significant upward trend in the cervical spinal cord (*F*_(3,10)_ = 2.732, *p* = 0.0997), with steady increased observed through day 7 (Figure 3E). In contrast, the thoracic spinal cord exhibited significant upregulation of *PGC-1α* mRNA (*F*_(3,11)_ = 20.45, *p* < 0.0001), demonstrating progressive restoration over time (Figure 3F). However, no statistically significant changes in *PGC-1α* mRNA levels were detected in the lumbar spinal cord (*F*_(3,11)_ = 1.808, *p* = 0.2039) (Figure 3G).

Since BMD-001S restored *PGC-1α* mRNA levels through regulation of its target, miR-485-3p, we examined the changes in PGC-1α protein expression across spinal cord regions to confirm its functional relevance. In the cervical region, the protein levels followed a pattern consistent with mRNA expression (*F*_(3,8)_ = 7.925, *p* = 0.0088) (Figure 3H). In contrast, the thoracic and lumbar regions exhibited trends that differed from their respective mRNA profiles (Figure 3I,J). In the thoracic spinal cord, PGC-1α protein expression did not reflect the upregulation observed at the mRNA level (*F*_(3,8)_ = 2.794, *p* = 0.1090), while the lumbar spinal cord exhibited day-dependent increase in the PGC-1α protein levels (*F*_(3,8)_ = 14.08, *p* = 0.0015) (Figure 3I,J).

### 2.6. BMD-001S Reduced CSF Neurofilament Light Chain Levels in SOD1^G93A^ Transgenic Mice

**Figure 4:**
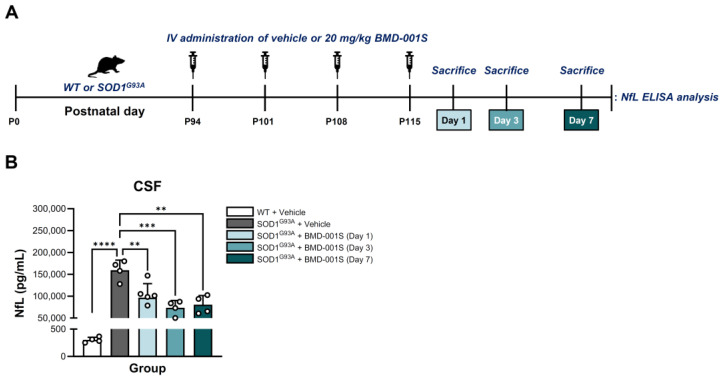
Evaluation of NfL concentration changes in CSF of SOD1^G93A^ transgenic mouse model. (**A**) Schematic procedure to measure NfL concentration in CSF in SOD1^G93A^ mouse model. (**B**) NfL variation was validated by ELISA. Data are presented as mean ± SEM. ** *p* < 0.01, *** *p* < 0.001, **** *p* < 0.0001 vs. vehicle-treated WT or SOD1^G93A^ group; n = 4–5 per group.

Elevated NfL levels in cerebrospinal fluid (CSF) are considered as a potential biomarker for ALS, reflecting ongoing neuronal damage and serving as a sensitive indicator of neuroaxonal injury in the disease [39,40,41]. We performed NfL enzyme-linked immunosorbent assay (ELISA) analysis to measure NfL concentrations in the CSF after the final IV administration of BMD-001S (Figure 4A). The results showed that SOD1^G93A^ transgenic mice exhibited significantly elevated CSF NfL levels compared to the age-matched WT controls. Additionally, the treatment with BMD-001S resulted in an approximately 50% decrease in CSF NfL concentrations relative to the vehicle-treated SOD1^G93A^ controls. This reduction was sustained for up to 7 days following the final BMD-001S administration (*F*_(4,17)_ = 25.04, *p* < 0.0001) (Figure 4B).

### 2.7. BMD-001S Improved Neuromuscular Function in SOD1^G93A^ Transgenic Mice

**Figure 5:**
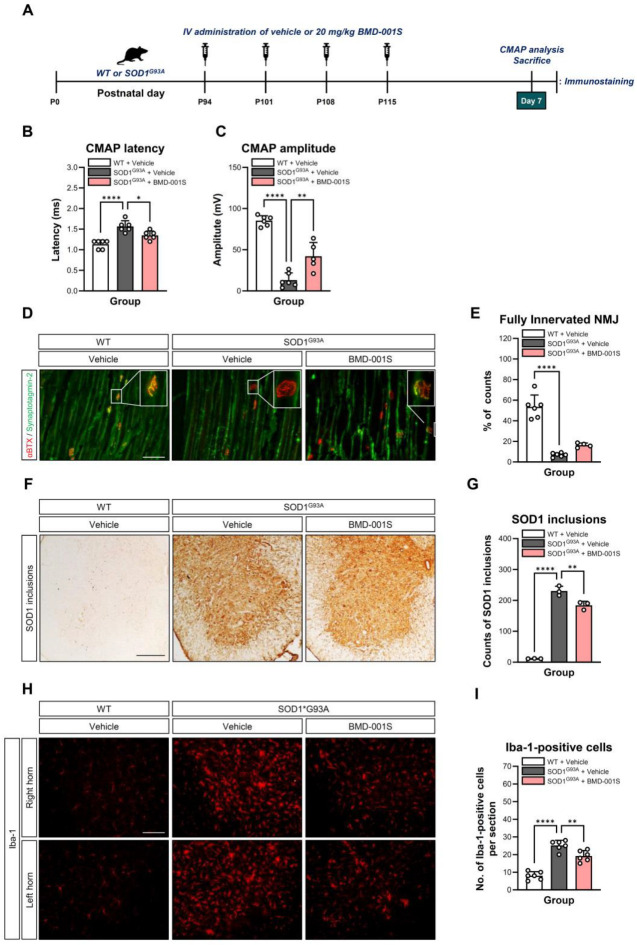
Neuroprotective effects of BMD-001S in SOD1^G93A^ transgenic mouse model. (**A**) Schematic procedure to evaluate neuroprotective effects of BMD-001S in SOD1^G93A^ mouse model. (**B**,**C**) Changes in CMAP latency and amplitude of hindlimbs after 20 mg/kg BMD-001S IV administration. (**D**) Representative images showing fully innervated NMJs in the gastrocnemius muscle. Presynaptic terminals (green) and postsynaptic endplates (red) are shown, with colocalization indicated by merged yellow signals. Scale bar, 100 μm. (**E**) Ratio of fully innervated NMJs in gastrocnemius muscles of WT and SOD1^G93A^ mouse model. (**F**) Representative microscopic images showing SOD1 inclusions in the ventral horn of the lumbar spinal cord of WT and SOD1^G93A^ mouse models. Scale bar, 200 μm. (**G**) Comparison of total SOD1 inclusion counts observed by a microscope. (**H**) Representative immunohistochemical images of Iba1 immunoreactivity (red) in the lumbar spinal cord of WT and SOD1^G93A^ mouse model. Scale bar, 100 μm. (**I**) Comparison of total Iba1 counts observed by immunofluorescence microscopic observation. Data are presented as mean ± standard error of the mean. * *p* < 0.05, ** *p* < 0.01, **** *p* < 0.0001 vs. the vehicle-treated WT or SOD1^G93A^ group; n = 3–6 per group.

Although SOD1^G93A^ transgenic mice typically begin to exhibit overt disease symptoms after approximately 90 days of age, several studies [34,35] have reported early pathological features—such as motor neuron degeneration, muscle denervation, neuromuscular junction (NMJ) denervation, mitochondrial abnormalities, and white matter abnormality in the spinal ventral horn—as early as 40 days of age. To evaluate the functional integrity of motor neurons and neuromuscular transmission [42], we conducted compound motor action potential (CMAP) analysis at 7 days after the final administration of BMD-001S using 116-day-old mice (Figure 5A). BMD-001S treatment significantly reduced CMAP latency from 1.6 ms in the vehicle-treated SOD1^G93A^ mice to 1.4 ms, representing an approximate 15.5% improvement (*F*_(2,15)_ = 20.95, *p* < 0.0001) (Figure 5B). In terms of CMAP amplitude, the vehicle-treated SOD1^G93A^ mice showed a marked reduction to approximately 12 mV, or 15% of the 80 mV observed in WT controls. However, BMD-001S administration significantly restored the amplitude to approximately, 40 mV, a 3.2-fold increase compared to vehicle-treated SOD1^G93A^ mice (*F*_(2,14)_ = 65.05, *p* < 0.0001) (Figure 5C).

To determine whether these functional improvements were accompanied by preservation of NMJs, we performed immunofluorescent staining of the gastrocnemius muscles and quantified the percentage of fully innervated NMJs. One-way ANOVA revealed a significant overall group effect on NMJ innervation (*F*_(2,13)_ = 66.68, *p* < 0.0001). In post hoc analyses, BMD-001S–treated SOD1^G93A^ mice showed a trend toward an increased proportion of fully innervated NMJs, with an approximately two-fold increase compared to vehicle-treated SOD1^G93A^ controls (*p* = 0.0686).

BMD-001S-treated SOD1^G93A^ mice exhibited a trend toward an increased proportion of fully innervated NMJs, showing an approximate 2-fold improvement relative to vehicle-treated SOD1^G93A^ controls (*F*_(2,13)_ = 66.68, *p* < 0.0001) (Figure 5D,E).

### 2.8. BMD-001S Attenuates SOD1 Aggregation and Microglial Activation in the Lumbar Spinal Cord

Mutant SOD1 proteins in ALS are prone to misfolding and aggregation, forming cytoplasmic inclusions that induce mitochondrial dysfunction and neuroinflammation, contributing to motor neuron degeneration [10,43,44]. To investigate the pathological hallmarks associated with ALS progression, we assessed the levels of misfolded SOD1 inclusions and microglial activation in the lumbar spinal cord of SOD1^G93A^ transgenic mice using immunostaining analysis. In the BMD-001S-treated SOD1^G93A^ transgenic mice, treatment with BMD-001S significantly reduced the elevated SOD1 inclusions in the lumbar spinal cord, as compared to the vehicle-treated SOD1^G93A^ transgenic mice (*F*_(2,6)_ = 288.4, *p* < 0.0001) (Figure 5F,G).

In addition to protein aggregation, neuroinflammation—primarily mediated by activated microglia—is a key contributor to ALS pathology. To evaluate this aspect, we examined ionized calcium-binding adapter molecule 1 (Iba1) expression, a marker of microglial activation. The results showed that the elevated level of Iba1 in microglial cells due to ALS pathology was reduced by approximately 23% following BMD-001S IV administration, compared to the vehicle-treated SOD1^G93A^ transgenic mice with comparable disease severity (*F*_(2,15)_ = 59.06, *p* < 0.0001) (Figure 5H,I).

## 3. Discussion

The *SOD1*^G93A^ mutation is one of the major causative variants of familial ALS, driving toxic gain-of-function effects that promote motor neuron degeneration through mitochondrial dysfunction and SOD1 protein misfolding/aggregation, which disrupt cellular homeostasis [45,46]. The pathological features observed in SOD1-mutnat animal models suggest that, beyond familial ALS, similar mechanisms of motor neuron degeneration and diverse pathogenic cell-autonomous and non-cell-autonomous toxicity are also present in sporadic ALS. These findings suggest that some aspect of pathomechanisms identified in transgenic models may apply to understanding the cell death mechanism of sporadic ALS, with important implications for developing therapeutic strategies [47]. miRNAs are increasingly recognized as key regulators in ALS pathophysiology, including mitochondrial integrity and neuroinflammation, with miR-155 and miR-206 demonstrating pivotal roles in SOD1^G93A^ models [48,49,50,51]. In this study, *SOD1*^G93A^ mutation significantly upregulated miR-485-3p expression in HMC3 microglial cells. Clinical evidence implicates miR-485 dysregulation in neurodegenerative disease (e.g., AD and PD) through pathways involving neuroinflammation, apoptosis, and synaptic function [22,23,52], supporting its relevance in SOD1^G93A^-induced ALS pathology. Importantly, PGC-1α, a transcriptional coactivator regulating mitochondrial biogenesis and oxidative stress responses and considered a putative target of miR-485-3p, has been shown in previous cancer studies to directly bind miR-485-3p via its 3′-UTR [24,26]. Our results revealed significant downregulation of *PGC-1α* mRNA in SOD1^G93A^-expressing HMC3 cells compared to the SOD1^WT^ controls. Together, these findings suggest that SOD1^G93A^ expression is associated with miR-485-3p upregulation and concomitant reduction in PGC-1α expression in glial cells, functionally extending the relevance of this regulatory axis to an ALS-related cellular context.

Longitudinal studies have identified distinct temporal expression patterns of miRNAs during ALS. For instance, miR-206 and miR-199a-5p exhibit significant upregulation in early-stage ALS, while miR-133a demonstrates diagnostic significance in moderate-to-severe stages [53]. Recent data suggest that plasma miR-214 could predict the prognosis and disease progression of ALS, which was well correlated with NfL level [54]. In this study, we observed stage-specific dysregulation of miR-485-3p in SOD1^G93A^ transgenic mice, which significant upregulation in the spinal cords at late-stage disease. This temporal expression pattern suggests miR-485-3p contributes to ALS symptom exacerbation and may serve as a biomarker for disease progression. Furthermore, the association between miR-485-3p elevation and disease stage implicates its roles in modulating pathological mechanisms underlying disease progression, particularly in the context of *SOD1*^G93A^ mutation-induced pathology.

Cytotoxicity assessment is essential for evaluating nanoparticle safety and biocompatibility, critical determinants for therapeutic applications [55,56]. Nanoparticle characteristics—including size, shape, chemical composition, and surface charge—influence cellular membranes interactions that may disrupt membrane integrity and damage lipids, protein, and DNA [57]. Our previous study demonstrated that the BDDS™-based ASO-loaded poly-*L*-lysine-PEG nanoparticles exhibited no cytotoxicity at concentrations up to 2000 nM in GL261 cells [58]. In this study, BMD-001S treatment at concentrations exceeding 1000 nM reduced cell viability by approximately 18%, with only marginal cytotoxicity (~6%) at 2000–5000 nM. According to FDA guidance (ISO 10993-5) [59], these results indicate that BMD-001S treatment did not induce significant cytotoxicity in SOD1^G93A^-expressing HMC3 cells. In addition, BMD-001S treatment at lower concentrations of 10–100 nM significantly attenuated miR-485-3p overexpression and reversed *PGC-1α* mRNA downregulation in a dose-dependent manner. This demonstrates BMD-001S was efficiently internalized into cells and exerts pharmacodynamic activity, effectively modulating the miR-485-3p/PGC-1α levels in microglial cells. Taken together, these findings suggest that BMD-001S could be a safe and effective therapeutic treatment for ALS by low cytotoxicity with targeted efficacy against PGC-1α deficiency in microglial cells.

In the development of ALS therapeutics, one of the fundamental challenges is the effective delivery of therapeutic drugs to the central nervous system. The blood–brain barrier (BBB) and the blood-spinal cord barrier (BSCB) significantly restrict most drugs access to the motor neurons in the brain and spinal cords [60,61]. Therefore, efficient strategies to penetrate these barriers are essential for effective therapeutic development in ALS [61,62,63]. Several approved drugs, such as riluzole and edaravone have shown therapeutic effects on motor function by modulating oxidative stress, excitotoxicity, and mitochondrial dysfunction [64,65,66]. In our previous study, we demonstrated that IV administration of a LAT1-targeting nanoparticle formulation (ASO-BDDS™) efficiently penetrated the BBB, delivered ASOs to the brain, and downregulated its target miRNA expression [58], thereby providing supportive evidence consistent with LAT1-mediated nanoparticle uptake and transport. In this study, consistent with our previous findings, IV administration of 20 mg/kg BMD-001S significantly reduced SOD1^G93A^-induced miR-485-3p overexpression across cervical-, thoracic- and lumbar spinal cords compared to the vehicle-treated SOD1^G93A^ mice. *LAT1* mRNA and LAT1 protein have been reported to be highly expressed in the primary-cultured rat spinal cord endothelial cells and the BSCB of rats [67,68], supporting the likelihood of LAT1-mediated delivery. Taken together, these findings suggest that the BMD-001S are likely delivered to the spinal cord, at least in part via LAT1-mediated endocytosis or transcytosis, and are associated with reduced miR-485-3p expression in SOD1^G93A^ transgenic mice. Along with the decrease in miR-485-3p, we observed an increase in both *PGC-1α* mRNA and PGC-1α protein levels in the spinal cord, although the degree of change varied by region. This regional variability may be attributed to differences in translational efficiency or post-transcriptional regulatory mechanisms, which could lead to discrepancies between *PGC-1α* mRNA and PGC-1α protein levels [69]. In addition, PGC-1α protein stability and turnover can be regulated by proteasomal degradation pathways, and baseline molecular heterogeneity across spinal cord segments may further contribute to discordance between transcript and protein levels [70,71]. Taken together, these findings suggest that BMD-001S may serve as a promising candidate to improve PGC-1α deficiency by modulating miR-485-3p dysregulation in a region-specific manner within the spinal cord during ALS progression caused by *SOD1*^G93A^ mutation.

Mutations in SOD1 gene are known to induce the formation of SOD1 protein inclusions, which contribute to chronic activation of microglia. This sustained microglial activation triggers a persistent neuroinflammatory response, thereby exacerbating motor neuron degeneration in ALS [43,44,72]. Therefore, the clearance of SOD1 aggregates and reduction in neuroinflammation are considered critical strategies for prolonging survival and alleviating symptoms in ALS [10,43,44]. In this study, IV-delivered BMD-001S significantly reduced both SOD1 inclusions and Iba1 expression in the lumbar spinal cord of SOD1^G93A^ transgenic mice compared to the vehicle-treated SOD1^G93A^ controls. Paralleled to this reduction in pathological aggregates, the excessive NfL level in CSF of SOD1^G93A^ transgenic mice was significantly decreased after BMD-001S treatment, indicating mitigation of axonal degenerations [39,40,41]. Given the observed increase in PGC-1α expression following BMD-001S treatment, these findings suggest that BMD-001S may exert anti-inflammatory and neuroprotective mechanisms by enhancing PGC-1α activity [25], thereby facilitating clearance of toxic SOD1 aggregates and reducing neurodegeneration in the spinal cord of SOD1^G93A^-induced ALS. CMAP measurement and assessment of fully innervated NMJs are widely used in ALS research to evaluate motor neuron health and the integrity of the motor neuron-muscle connectivity [42]. In this study, IV administration of BMD-001S significantly attenuated SOD1^G93A^-induced abnormalities in electrophysiological parameters and enhanced NMJs innervation in the spinal cord. These findings indicate that BMD-001S not only facilitates effective neuromuscular signal conduction, but also contributes to the structural maintenance of NMJs, highlighting its potential as a promising neuroprotective effect at both functional and molecular levels for ALS therapy. Nevertheless, the present study primarily focused on microglial cells and did not address the direct neuronal effects of BMD-001S. Further studies are needed to investigate cell-type–specific uptake and efficacy of BMD-001S to elucidate its mechanisms of action at both functional and molecular levels.

## 4. Materials and Methods

### 4.1. Generation of Stable SOD1^WT^ or SOD1^G93A^-Expressing HMC3 Cell Lines

The human microglial cell line HMC3 was used to establish stable cell lines expressing either *SOD1*^WT^ or *SOD1*^G93A^. HMC3 cells were cultured in Dulbecco’s Modified Eagle Medium (DMEM) supplemented with 10% fetal bovine serum and 1% penicillin, in a 95% O_2_/5% CO_2_ incubator at 37 °C. Cells were transfected with either pCDH-EF1α-SOD1^WT^-AcGFP-T2A-Puro or pCDH-EF1α-SOD1^G93A^-AcGFP-T2A-Puro using JetOPTIMUS^®^ DNA transfection reagent (Cat #101000051, Polyplus, Illkirch, France) according to the manufacturer’s instruction. Detailed sequences of SOD1^WT^-AcGFP and SOD1^G93A^-AcGFP expression vectors are provided in Supplementary Figure S1A,B. Forty-eight hours after transfection, puromycin (Cat #A1113802, Thermo Fisher Scientific, Waltham, MA, USA) were added to the cells at a concentration of 0.5 μg/mL. The concentration was gradually increased to 10 μg/mL over a period of 15 days to select for stably transfected cells. Expression of SOD1^WT^-AcGFP and SOD1^G93A^-AcGFP fusion proteins in HMC3 cells were confirmed by fluorescence microscopy (Supplementary Figure S1C). GFP-positive cells were further purified by fluorescence-activated cell sorting (FACS) using a BD FACSMelody^TM^ Cell Sorter (BD Biosciences, San Jose, CA, USA).

### 4.2. Animals

Male SOD1^G93A^ transgenic mice expressing mutant human SOD1*^G93A^* (hemizygous Tg(SOD1^G93A^)1Gur) and WT littermates were used in the study (B6SJL-Tg(SOD1^G93A^)1Gur/J, Strain #002726, Jackson Laboratory, Bar Harbor, ME, USA) [33]. Only male mice were used to reduce experimental variability associated with sex-dependent factors. A total of 50 SOD1^G93A^ transgenic and 20 WT mice were used in all experiments. All animal procedures were carried out according to Korea Food and Drug Administration guidelines for the care and use of laboratory animals, approved by the Institutional Animal Care and Use Committee (IACUC) of KBIO Health (approval number: KBIO-IACUC-2022-141, 22 August 2022), with drug administration and in-life biological sampling conducted at Naason Science Inc. (Cheongju-si, Republic of Korea). Mice were housed under controlled environmental conditions: temperature of 22 ± 1 °C, relative humidity 30–50%, and under 12 h light/dark cycles. All mice were housed in cages with clean bedding covering the ground that were changed as frequently as needed, to provide the animals with dry bedding. This basic environment was enriched with the addition of play tunnels or igloos and wooden nesting material. Food and water were available ad libitum to the mice in their home cages. The WT and transgenic mice were randomly allocated to each experimental group (n = 3–5 per group). No animals were excluded from the experiments due to abnormal body weight loss, death, or other specific issues during the housing, treatment, or maintenance periods.

### 4.3. Experimental Design, Randomization, and Blinding

The study comprised five sequential experimental components designed to evaluate the biological relevance of miR-485-3p, as well as the safety, efficacy, and therapeutic effects of BMD-001S in ALS models, as follows: (1) in vitro and in vivo characterization of SOD1^G93A^ and miR-485-3p expression; (2) in vitro assessment of the cytotoxicity and efficacy of BMD-001S; (3) evaluation of the biomolecular therapeutic effects of BMD-001S in the spinal cords of SOD1^G93A^ transgenic mice; (4) analysis of NfL levels in CSF as a pharmacodynamic biomarker; and (5) assessment of functional and pathological outcomes, including CMAP, NMJ integrity, SOD1 protein inclusions, and neuroinflammation.

To ensure appropriate randomization and minimize potential bias, the experiments were conducted as follows. WT and SOD1^G93A^ transgenic mice were randomly assigned to experimental groups (n = 3–5 per group), and potential confounders were minimized through randomization and balanced group allocation. Animal handling, dosing, tissue and CSF sampling, CMAP measurements, as well as NMJ and Iba1 immunostaining, were performed by an independent contract research organization (CRO; Naason Science, Republic of Korea). The NfL ELISA analyses were conducted by a separate CRO (JS Link, Seoul, Republic of Korea), while in vitro experiments, qPCR analyses, PGC-1α ELISA, and SOD1 immunostaining were performed at BIORCHESTRA Co., Ltd. (Daejeon, Republic of Korea). Although blinding was not feasible during multi-step experimental procedures, objective quantitative endpoints were employed, and data analysis was conducted in a blinded manner using coded datasets.

### 4.4. Target Gene Knockdown Analysis

#### 4.4.1. RNA Extraction

Total RNA, including both mRNA and enriched miRNA, was isolated from HMC3 cells and mouse spinal cords tissues using the Maxwell^®^ RSC miRNA Tissue kit (Cat #AS1460, Promega, Madison, WI, USA) according to the manufacturer’s instructions. The concentration and purity of the extracted RNAs were determined with a Nanodrop ONE UV spectrophotometer (DeNovix, Wilmington, DE, USA). Only samples with an optimal density 260/280 ratio between 1.8 and 2.0 were used for further analysis.

#### 4.4.2. qPCR Analysis

cDNA for microRNA was synthesized from isolated RNA using the microRNA RT Kit (Cat #MMR-003, Enzynomics, Daejeon, Republic of Korea), while cDNA for mRNA was generated using the TOPscript™ RT DryMIX; dN6 plus (Cat #RT210, Enzynomics, Daejeon, Republic of Korea), both according to the manufacturers’ instructions.

Relative expression levels of miR-485-3p were quantified using the CFX Connect Real-Time PCR Detection System (Cat #1855201, Bio-Rad, Hercules, CA, USA) with TOPreal™ qPCR 2X PreMIX (Cat #RT620M, Enzynomics, Daejeon, Republic of Korea) and TaqMan MGB Probe (Cat #4316032, Applied Biosystems, Foster City, CA, USA). The qPCR protocol consisted of an initial denaturation at 95 °C for 10 min, followed by 40 cycles of 95 °C for 10 s, 58 °C for 1 min, and 72 °C for 10 s. FAM fluorescence was measured at each annealing step. Final expression levels were calculated using the 2^−ΔΔCt^ method, with U6 (for human samples) or miR-6366 (for mouse samples) serving as internal controls for miR-485-3p quantification, and were expressed as fold change relative to the control group [22].

Relative mRNA expression levels of PGC-1α were also determined using the CFX Connect Real-Time PCR Detection System (Cat #1855201, Bio-Rad, Hercules, CA, USA) with TOPreal™ SYBR Green qPCR PreMIX (Cat #RT500M, Enzynomics, Daejeon, Republic of Korea). The reaction conditions included an initial denaturation at 95 °C for 10 min, followed by 40 cycles of 95 °C for 10 s, 56 °C for 10 s, and 72 °C for 30 s. SYBR Green fluorescence was detected at each extension step. Final expression levels were calculated by the 2^−ΔΔCt^ method with 18S rRNA serving as an internal control for *PGC-1α* mRNA expression and were expressed as fold change relative to the control group [73]. All primer sequences for analyzing miRNA and mRNA used in this study are listed in Supplementary Tables S2 and S3.

### 4.5. Preparation of BMD-001S

BMD-001S, a nanoparticle formulation designed for the delivery of an antisense oligonucleotide (ASO) targeting miR-485-3p, was prepared using a protocol adapted from our previous study, in which this formulation strategy was validated [58]. The chemically modified ASO sequence (5′-AGAGAGGAGAGCCGUGUAUGAC-3′), which has been previously shown to achieve efficient target knockdown, was purchased from GeneDesign, Inc. (Osaka, Japan). Briefly, the ASO was dissolved in nuclease-free water at a concentration of 180 μM. The BMD-001S nanoparticles were generated by complexing the ASO with MeO-PEG-PLL(NA/MPA), a non–LAT1-targeted copolymer, and Phe-PEG-PLL(NA/MPA), a LAT1-targeted copolymer, as described in our prior work [58].

The polymer solution was formulated by dissolving LAT1-targeted polymer and non-target polymer at 10 mg/mL in 100 mM sodium phosphate buffer (pH 7.4), mixed at a 1:3 (*v*/*v*) ratio. This mixture was further diluted to a final polymer concentration of 6.4 mg/mL using 800 mM dithiothreitol in 100 mM phosphate buffer. The BMD-001S were formed by combining the ASO and polymer solutions at a 2:1 (*v*/*v*) ratio using a microfluidic mixer (Blaze, Precision NanoSystems [Cytiva], Vancouver, BC, Canada), as previously described [58]. Following mixing, the formulation was incubated at 25 °C for 30 min to allow nanoparticle stabilization. The resulting nanoparticle solution was purified by dialysis (molecular weight cut-off: 20 kDa, Thermo Fisher Scientific, Haverhill, MA, USA) against 0.5% DMSO in 5 mM phosphate buffer for one day, followed by dialysis in 5 mM phosphate buffer for two additional days. The purified BMD-001S was then filtered through a 0.22 μm polyethersulfone bottle-top filter (Corning^®^, Glendale, AZ, USA) and lyophilized with cryoprotectants (mannitol and sucrose, 1:4 *v*/*v*). The final N-to-P ratio (the ratio of amine groups from the polymer to phosphate groups from the ASO) was set at 1.4. Lyophilized BMD-001S was stored at −20 °C until use.

Particle size distribution, PDI, and zeta potential were measured by dynamic light scattering using a Zetasizer (Ultra Red, Malvern Panalytical, Malvern, UK). EE% and ASO concentration were quantified by high-performance liquid chromatography using an Infinity II 1260 system (Agilent Technologies, Santa Clara, CA, USA) under the following conditions: solvent A, 10 mM NaOH in 30% methanol; solvent B, 1 M NaClO_4_ with 10 mM NaOH in 30% methanol; flow rate, 0.8 mL/min. For analysis, 10 μL of 10 μM BMD-001S solution was injected into a BioPro IEX QF column (5 μm, 100 × 4.6 mm, YMC, Kyoto, Japan) at 60 °C. The identical BMD-001S formulation prepared as described above was used for all subsequent in vitro and in vivo experiments.

### 4.6. Cell Viability and Cytotoxicity Assays

Cell viability and cytotoxicity assays were performed as previously described [58]. Stably SOD1^G93A^-expressing HMC3 cells were used for these assays and maintained at 37 °C, under 95% O_2_ and 5% CO_2_ conditions using appropriate medium. The culture medium was replaced every 2 to 3 days. When cells reached approximately 80% confluence, the cells were sub-cultured using 0.05% trypsin-EDTA (Cat #15400054, Gibco, Waltham, MA, USA). For experiments, cells were seeded at a density of 1 × 10^4^ cells in 96-well culture plates (Cat #30096, SPL, Pocheon-si, Republic of Korea). Cells were treated with BMD-001S, dissolved in nuclease-free water, at concentrations of 5, 50, 500, 1000, 2000, and 5000 nM. After 48 h of treatment, cell viability was assessed using the Cellrix Viability Assay Kit (WST-8, MediFab, Seoul, Republic of Korea) according to the manufacturer’s instructions. Briefly, 10% Cellrix solution was added to each well, and the plates were incubated at 37 °C for 2 h. Absorbance was measured at 450 nm using a microplate reader (SpectraMax iD3; Molecular Devices, San Jose, CA, USA).

Cytotoxicity was evaluated using the CytoTox96^®^ Non-Radioactive Cytotoxicity Assay Kit (Cat #G1780, Promega, Madison, WI, USA), following the manufacturer’s protocol. Cell culture supernatants were collected and centrifuged at 250× *g* for 4 min. Then, 50 μL of the supernatant was transferred to a new 96-well microplate, followed by the addition of 50 μL CytoTox96^®^ Reagent. Plates were incubated at room temperature for 30 min, after which 50 μL of stop solution was added to each well. Absorbance was measured at 490 nm using the SpectraMax iD3 microplate reader within 1 h.

### 4.7. Intravenous Administration of BMD-001S in SOD1^G93A^ Transgenic Mice

BMD-001S was administered to SOD1^G93A^ transgenic mice beginning at 94 days postnatally, which corresponds to the onset of clinical disease symptoms (as evidenced by hindlimb tremor) in this model [34,35]. The dose of BMD-001S (20 mg/kg) was determined based on our previous study using the same ASO-loaded nanoparticles, in which intravenous administration at this dose achieved efficient target knockdown [58]. The prepared BMD-001S working solution, dissolved in 0.9% sterile physiological saline for intravenous (IV) injection, was administered at a dose of 20 mg/kg, once weekly for four consecutive weeks. In this regimen, intravenous injections were performed on postnatal days P94, P101, P108, and P115. Throughout the treatment period, no deaths were observed among the BMD-001S treated animals.

### 4.8. Tissue and Sample Collection

#### 4.8.1. Timeline for Tissue and Sample Collection

Prior to tissue collection, mice were deeply anesthetized with a mixture of Zoletil and Rompun and were perfused transcardially with 0.9% saline to ensure thorough removal of blood from all tissues. SOD1^WT^ and vehicle-treated SOD1^G93A^ control groups were sacrificed at 115 days of age, corresponding to the final injection time point of BMD-001S administration in SOD1^G93A^ transgenic mice. The SOD1^G93A^ transgenic mice treated with BMD-001S were sacrificed at 1, 3, and 7 days after the final injection. Spinal cord tissues for qPCR and ELISA analyses, as well as CSF samples for NfL quantification, were collected at each of these time points. For histological analyses, gastrocnemius muscle and spinal cord tissues were sampled specifically at 7 days after the final BMD-001S administration.

#### 4.8.2. Spinal Cord Tissues Collection for qPCR and ELISA Analysis

Spinal cord tissues were harvested and immediately placed in microcentrifuge tubes containing 1 mL of PBS and lysing beads. The tissues were homogenized using a TissueLyser II (Qiagen, Hilden, Germany), then incubated on ice for 30 min to ensure complete lysis. The homogenates were centrifuged at 15,000 rpm for 30 min at 4 °C. The resulting supernatants were carefully collected and divided into two equal portions: one portion was used for RNA extraction and subsequent qPCR analysis, while the other was reserved for protein extraction and ELISA analysis. Spinal cords with insufficient tissue size were subjected to either qPCR or ELISA analysis only or were excluded from further analyses.

#### 4.8.3. CSF Collection

CSF samples were collected from the cisterna magna as previously described [74], immediately after anesthesia and prior to perfusion. The area was surgically exposed, and CSF was carefully aspirated using a fine glass capillary or syringe, ensuring minimal blood contamination. Collected CSF samples were immediately placed on ice and stored at –80 °C until further analysis.

### 4.9. Gastrocnemius Muscle and Lumbar Spinal Cord Tissue Collection for Histological Analysis

For histological analysis, both gastrocnemius muscle and lumbar spinal cord tissues were harvested 7 days after the final administration of BMD-001S. Muscle and lumbar spinal cord samples were fixed in 4% paraformaldehyde (Cat #PC2205-100-74, Biosesang, Yongin-si, Republic of Korea) for 24 h at 4 °C. After fixation, the tissues were sequentially cryoprotected by immersion in 15% sucrose solution in PBS until submerged, followed by overnight incubation in 30% sucrose solution in PBS. The cryoprotected tissues were embedded in OCT compound to prepare frozen blocks suitable for sectioning and immunostaining.

### 4.10. ELISA

#### 4.10.1. Quantification of PGC-1α Protein Levels in the Spinal Cord Tissues

Spinal cord tissue lysates were diluted 1:2 prior to both total protein quantification and PGC-1α ELISA analysis. PGC-1α protein levels were measured using a mouse-specific ELISA kit (Cat #E1487Mo, BTLab, Shanghai, China) in accordance with the manufacturer’s instruction. The concentration of PGC-1α in each sample was determined by comparison to a standard regression curve. Total protein concentration was measured using the DC Protein Assay (Cat #5000116, Bio-Rad, Hercules, CA, USA) with bicinchoninic acid standard curve. PGC-1α concentrations obtained from the ELISA (ng/L) were normalized to the total protein content (mg/L) for each sample, and final PGC-1α concentration was expressed in ng/mg.

#### 4.10.2. Quantification of NfL Protein Levels in CSF

NfL was therefore selected as the primary biomarker due to its established sensitivity and clinical relevance as an indicator of axonal damage and disease progression in ALS [75]. The collected CSF samples were diluted at ratios ranging from 1:400 to 1:1200 prior to NfL analysis. NfL protein levels were measured using the Simoa^®^ NF-Light^TM^ Advantage Kit HD-1 ELISA (Cat #103186, Quanterix, Billerica, MA, USA) following the manufacturer’s instruction. NfL concentrations were calculated based on a standard regression curve. The final NfL concentration was adjusted according to the dilution factor and was expressed in pg/mL.

### 4.11. CMAP Measurement

CMAP measurements were performed at the level of the sciatic nerve to assess nerve functionality following the established protocol [42]. Mice were anesthetized, and body temperature was maintained throughout the procedure. Electrical stimulation was applied to the sciatic nerve using percutaneous needle electrodes, while CMAPs were recorded from target muscles of both the forelimbs and hindlimbs using 27-gauge needle recording electrodes connected to the Ultra S Pro100 system (Natus, Middleton, WI, USA). Supramaximal stimulation was delivered by gradually increasing the stimulus intensity starting from 5.48 mA, corresponding to the minimum output of the instrument, up to a maximum of 9 mA, until a plateau in CMAP amplitude was observed. For each animal, three consecutive recordings were obtained, and the peak-to-peak amplitudes were measured. The average of these values was calculated and used as the final CMAP amplitude for each subject.

### 4.12. Immunostaining

#### 4.12.1. NMJ Staining

NMJ staining was performed as previously described, with minor modifications [76,77]. For the evaluation of NMJ and muscular innervation, immunohistochemical analysis was performed on gastrocnemius muscle sections using anti-synaptotagmin-2 (a pre-synaptic marker) and α-bungarotoxin (a post-synaptic marker). OCT-embedded gastrocnemius muscles were sectioned at a thickness of 20 µm and mounted onto microscope slides. Sections were washed with Tris-buffered saline (TBS), permeabilized in TBS with tween 20 (TBS-T) for 15 min, rinsed twice in BS, and then blocked in TBS containing 5% normal goat serum and 0.2% Triton X-100 for 1 h at room temperature (RT). After blocking, sections were incubated overnight at 4 °C with anti-synptotagmin-2 and anti-α-bungarotoxin antibodies. The following day, sections were washed in TBS and incubated with appropriate secondary antibodies for 1 h at RT. After a final wash in TBS, the sections were dried and mounted with a DAPI-containing mounting medium. NMJs were visualized using a Leica Thunder confocal microscope (Leica Microsystems, Wetzlar, Hesse, Germany). For analysis, regions showing complete overlap of anti-synaptotagmin-2 and α-bungarotoxin staining were classified as ‘fully innervated’ NMJs. For each mouse, 2–3 non-adjacent gastrocnemius muscle sections were analyzed, and a minimum of 30 NMJs per animal were randomly selected across sections. Image analysis was performed by defining regions of interest (ROIs) around individual NMJs, and consistent fluorescence intensity thresholding was applied across all images to ensure objective and reproducible quantification.

#### 4.12.2. SOD1 Inclusion and Iba1 Immunostaining

Lumbar spinal cord sections (20 μm thickness) were first processed for SOD1 inclusion analysis. After washing in 0.1 M phosphate-buffered saline (PBS) (Thermo Fisher Scientific, Waltham, MA, USA), sections were blocked with 0.5% bovine serum albumin (BSA) (Sigma-Aldrich, St. Louis, MO, USA) and 0.1% Triton X-100 (Sigma-Aldrich, St. Louis, MO, USA). Sections were then incubated at 4 °C for 2 days with a primary antibody against misfolded SOD1 (clone B8H10; 1:500; MEDIMABS, Montreal, QC, Canada). Detection was performed using a biotin-conjugated secondary antibody (Vector Laboratories, Newark, CA, USA), followed by the VECTASTAIN^®^ ABC Kit (Vector Laboratories, Newark, CA, USA) and DAB substrate (Vector Laboratories, Newark, CA, USA). After staining, sections were coverslipped with Permount™ Mounting Medium (Thermo Fisher Scientific, Waltham, MA, USA) and imaged using a Leica bright-field microscope (Leica Microsystems, Wetzlar, Hesse, Germany). SOD1 inclusions were quantified using ImageJ software (Version 1.52a, National Institutes of Health, Bethesda, MD, USA).

For microgliosis analysis, adjacent sections were stained with an anti-Iba1 antibody (1:2000; Wako Pure Chemical Industries, Osaka, Osaka Prefecture, Japan) following similar blocking and incubation procedures as described for NMJ staining. After incubation with an Alexa Fluor 594-conjugated secondary antibody (Thermo Fisher Scientific), sections were imaged using an Olympus BZ-X710 fluorescence microscope (Olympus Corporation, Tokyo, Japan). Fluorescence intensity in the ventral horn was quantified using ImageJ software (National Institutes of Health, Bethesda, MD, USA).

### 4.13. Statistical Analysis

All values were expressed as mean ± standard error of the mean (SEM). All experiments using cell lines were repeated at least two or three times. The statistical significance of the values obtained across the two groups were determined using an unpaired *t*-test. Multiple comparisons among the groups were performed by one-way analysis of variance (ANOVA) followed by Tukey’s post hoc test. Asterisks *, **, ***, and **** in figures represent significance levels *p* < 0.05, <0.01, <0.001, and <0.0001, respectively. All statistical analyses were performed using GraphPad Prism 10 (version 10.3.0, GraphPad Software, Inc., San Diego, CA, USA).

## 5. Conclusions

This study demonstrates that miR-485-3p is markedly upregulated in the SOD1^G93A^ ALS model and contributes to disease progression by suppressing PGC-1α, a critical regulator of mitochondrial homeostasis and neuroinflammation. Systemic administration of BMD-001S, which encapsulates an ASO specifically targeting miR-485-3p, effectively reversed SOD1^G93A^-induced miR-485-3p upregulation and restored PGC-1α expression in both microglial cells and the spinal cords of the SOD1^G93A^ transgenic mice. BMD-001S treatment reduced SOD1 protein inclusions, microglial activation, and NfL levels in CSF, consistent with attenuation of neuroinflammation and axonal degeneration. Improvements in CMAP amplitude and NMJ integrity further support its neuroprotective potential. Altogether, these findings suggest that BMD-001S effectively modulates the miR-485-3p/PGC-1α axis and ameliorates key pathological features of ALS, representing its potential as a therapeutic strategy. Given that the present study focused on short-term post-treatment outcomes in male mice, further studies including long-term evaluation and female cohorts will be required to assess the pharmacokinetics, tissue distribution, dosing strategy and potential sex-specific differences in the therapeutic effects of BMD-001S in ALS.

## Data Availability

The original contributions presented in this study are included in the article/Supplementary Material. Further inquiries can be directed to the corresponding authors.

## Supplementary Materials

The following supporting information can be downloaded at: https://www.mdpi.com/article/10.3390/ijms27020615/s1.

## Abbreviations

The following abbreviations are used in this manuscript:

ALS	Amyotrophic lateral sclerosis
ANOVA	Analysis of variance
ASO	Antisense oligonucleotide
BBB	Blood–brain barrier
BSA	Bovine serum albumin
BSCB	Blood-spinal cord barrier
CMAP	Compound motor action potential
CNS	Central nervous system
CSF	Cerebrospinal fluid
DMEM	Dulbecco’s Modified Eagle Medium
EE	Encapsulation efficiency
ELISA	Enzyme-linked immunosorbent assay
FACS	Fluorescence-activated cell sorting
HMC3	Human microglial clone 3
IACUC	Institutional Animal Care and Use Committee
Iba1	Ionized calcium-binding adapter molecule 1
IV	Intravenous
LAT1	L-type amino acid transporter 1
NfL	Neurofilament light chain
NMJ	Neuromuscular junction
PBS	Phosphate-buffered saline
PDI	polydispersity index
PGC-1α	Peroxisome proliferator-activated receptor gamma coactivator 1-alpha
qPCR	Quantitative PCR
RT	Room temperature
SOD1	Superoxide dismutase 1
TBS	Tris-buffered saline
TBS-T	TBS with tween 20
WT	Wild-type
