# Supplementary material for: Modulation of the miR-485-3p/PGC-1α Pathway by ASO-Loaded Nanoparticles Attenuates ALS Pathogenesis
Source: Int J Mol Sci. 2026 Jan 7;27(2):615. doi: 10.3390/ijms27020615 (PMC12840692; doi:10.3390/ijms27020615)
Supplement: Supplementary file 1 [file ijms-27-00615-s001.zip › ijms-4056358-supplementary.pdf]

A

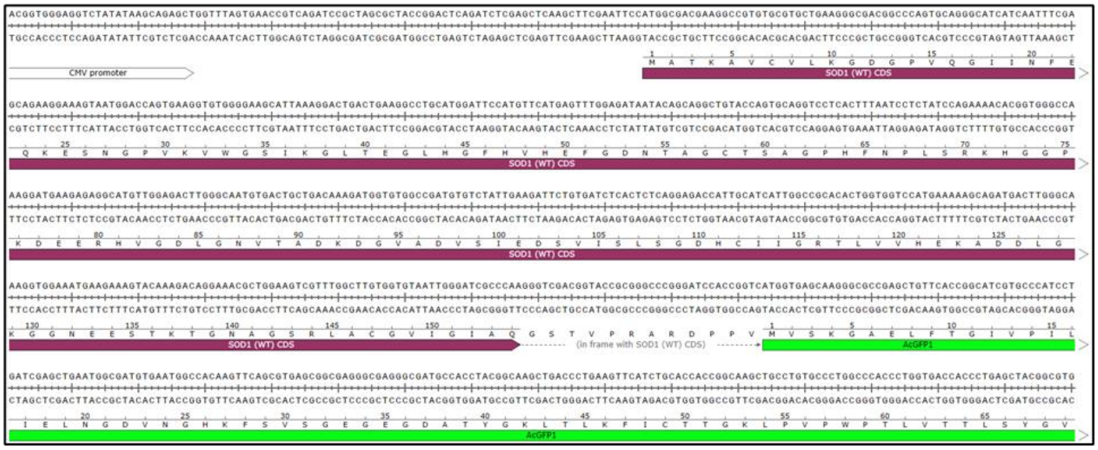

CMV promoter
SOD1 (WT) CDS
(in frame with SOD1 (WT) CDS)
AcGFP1

B

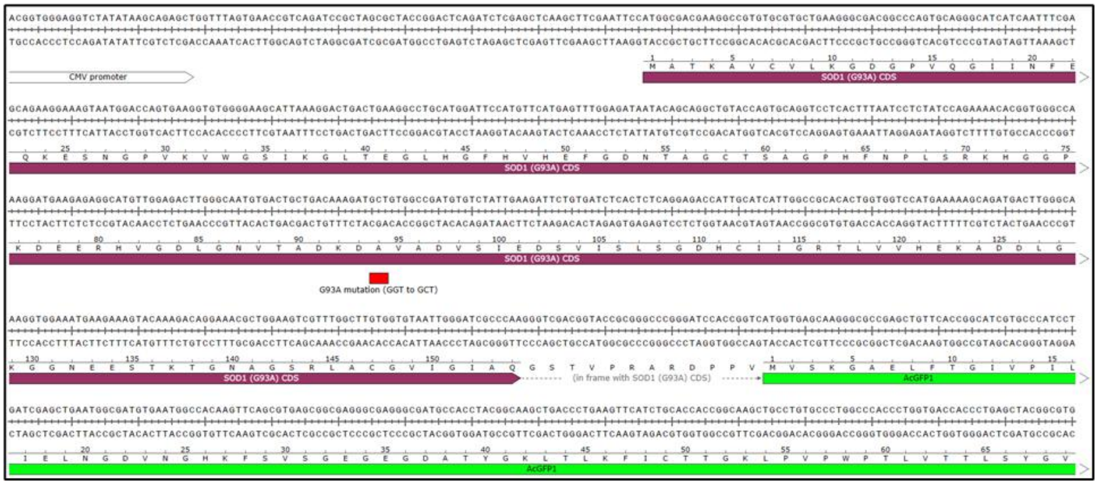

CMV promoter
SOD1 (G93A) CDS
G93A mutation (GGT to GCT)
(in frame with SOD1 (G93A) CDS)
AcGFP1

C

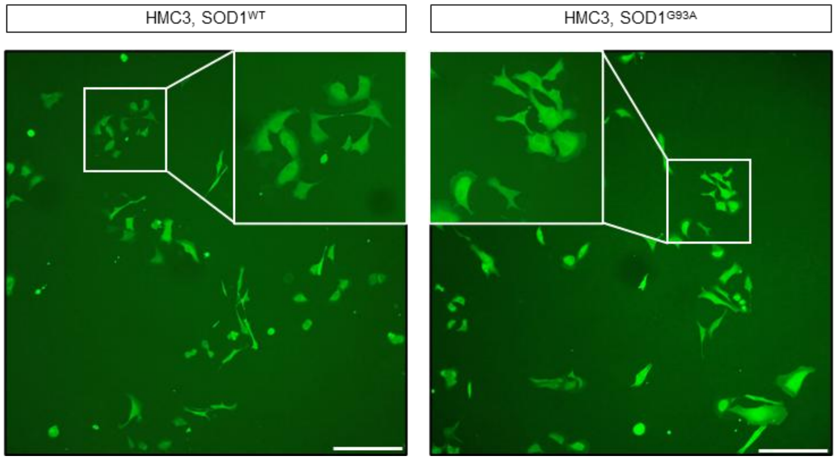

HMC3, SOD1^{WT}
HMC3, SOD1^{G93A}

Supplementary Figure S1. Confirmation of SOD1^{WT}- and SOD1^{G93A}-expressing cells.

(A) SOD1^{WT}-AcGFP and **(B)** SOD1^{G93A}-AcGFP expression vector sequence. The AcGFP nucleotide sequence was tagged to the 3' end of each SOD1 sequence. **(C)** Microscopic fluorescence observation of SOD1^{WT} and SOD1^{G93A} expressing cells. Scale bar, 100 μm.

Supplementary Table S1. Characterization of BMD-001S.

	Size (diameter, nm)	PDI	Zeta potential (mV)	pH	EE%
**BMD-001S**	44.6 ± 5.5	0.14 ± 0.04	8.3 ± 1.4	7.58 ± 0.13	98.40 ± 3.50

PDI, polydispersity index; EE%, encapsulation efficiency.

Supplementary Table S2. Primers for analyzing miRNA.

Name	N-term	Sequence	C-term	Length (nt)	Tm (℃)
miRNA-485-3p – Forward	-	5′-GTCATACACGGCTCTCCTCTCTAA-3′	-	24	60.2
miRNA-6366 – Forward	-	5’-AGCTAAGGGGCCCGGGGAGCCA-3’	-	22	70.9
RNU6 – Forward	-	5’- GCTTCGGCAGCACATATACTAAAAT-3’	-	25	54.4
URP3	-	5’-GAATCGAGCACCAGTTACG-3’	-	19	55.2
Probe	6-FAM	5′-CGAGGTCGACTTCCTAGA-3′	NFQ (MGB)	18	68.0

6-FAM, 5'-fluorescein phosphoramidite; NFQ, 3' nonfluorescent quencher; MGB, minor groove binder.

Supplementary Table S3. Primers for analyzing mRNA.

Name	Sequence	Length (nt)	Tm (°C)
Ppargc1α – Forward	5′-AACCAGTACAACAATGAGCCTG-3′	22	53.0
Ppargc1α – Reverse	5′-AATGAGGGCAATCCGTCTTCA-3′	21	52.4
18S rRNA – Forward	5’-GCAATTATTCCCCATGAACG-3’	20	49.7
18S rRNA – Reverse	5’- GGCCTCACTAAACCATCCAA-3’	20	51.8
